# Surgical androgen deprivation therapy in advanced prostate cancer in patients of African descent: comparison of biochemical efficacy of bilateral total and subcapsular orchidectomy

**DOI:** 10.4314/ahs.v23i1.50

**Published:** 2023-03

**Authors:** Abayomi K Arogundade, Ademola A Popoola, Abdulwahab A Ajape, Olajide O Abiola, Sikiru A Biliaminu

**Affiliations:** 1 Federal Teaching Hospital, Urology Unit, Department of Surgery; 2 University of Ilorin Teaching Hospital, Urology Division, Department of Surgery; 3 Bowen University Teaching Hospital, Urology Unit, Department of Surgery; 4 University of Ilorin Teaching Hospital, Chemical Pathology and Immunology

**Keywords:** androgen deprivation therapy, subcapsular orchidectomy, total orchidectomy

## Abstract

**Background:**

Surgical androgen deprivation therapy (ADT) to treat advanced prostate cancer can be achieved either by bilateral total orchidectomy (BTO) or bilateral subcapsular orchidectomy (BSCO). However, biochemical and clinical equivalence between BTO and BSCO among native Africans is undocumented.

**Objective:**

To compare the biochemical response (testosterone and prostate specific antigen) in patients who had BTO and BSCO for advanced prostate cancer.

**Methods:**

A randomized single- blind study of 64 consenting patients that underwent either BTO or BSCO. Pre- and post-operative PSA and testosterone assays were done serially at intervals and compared between each treatment group.

**Results:**

Each treatment group were similar with no statistically significant difference in terms of age (p= 0.449) or degree of tumor differentiation (p =0.714). Neither median testosterone (p= 0.515) nor the mean pre-operative PSA differ between the two groups (p = 0.482). Also, similar trends were noticed post operatively except at the 2^nd^ month when a statistically significant difference was recorded (p = 0.003).

**Conclusion:**

The two techniques of orchidectomy were effective in accomplishing androgen deprivation. They produced similar biochemical (testosterone and PSA) response.

## Introduction

Prostate cancer is the most commonly diagnosed cancer among Nigerian men^1^. In a more recent community-based screening in Nigeria, high prevalence was observed with majority already had advanced disease at the time of diagnosis^2^.

In many of the studies in Nigeria, late presentation with an advanced stage of the disease was the norm^3^–^7^. Due to the late presentation, treatment has been essentially palliative with androgen deprivation therapy (ADT) aimed at achieving castrate testosterone levels; i.e., (testosterone level of less than 50ng/dl or less than 5% of serum testosterone before castration)^8^,^9^. Androgen deprivation can be achieved either surgically (by a bilateral orchidectomy) or by medical therapy with (Luteinizing Hormone Releasing Hormone (LHRH) analogs/antagonists, anti-androgens, or oestrogens). Bilateral total orchidectomy (BTO), which involves total excision of the testes and epididymis, has been found to achieve castrate testosterone levels within 24 hours^10^. It is a one-time treatment and the least expensive with comparable efficacy to other modalities of treatment^10^,^11^. Despite this, most patients especially in developed countries, when given a choice prefer medical androgen ablation due to psychological effect of an empty scrotum and poor body image^12^,^13^.

Riba in 1942 described the technique of bilateral subcapsular orchidectomy (BSCO), which entails leaving the tunica albuginea and epididymis in the scrotum 14. This leaves behind palpable “testicles” and has helped to avoid some of the psychological effect of loss of testes (empty scrotum) following total orchidectomy 9. However, McDonald and Calams in 1959 showed that the tunica albuginea and epididymis when retained in a sub-capsular orchidectomy have Leydig-like cells which continue to produce testosterone thereby precluding complete androgen ablation at the testicular level 15. Also, O'Conor et al, in 1963 showed that human chorionic gonadotrophin stimulation in patients, who have undergone sub-capsular orchidectomy, produced an increase in urinary excretion of testosterone metabolic product (androsterone)^16^. These studies questioned the efficacy of BSCO; thus, BTO was promoted as the treatment of choice 17.

Recently, there has been a renewed interest in BSCO as a treatment option in advanced prostate cancer because of the observed persistent oncological effectiveness, and better psychological outcomes 18. Furthermore, some studies have also found significantly fewer post-operative complications with BSCO than BTO 13,19.

Demonstration of the biochemical response to either type of bilateral orchidectomy in native Africans are rather scanty in indexed publications. Furthermore, prostate cancer has a heterogeneous tumor presentation coupled with the significant racial differences in the incidence, prevalence, biologic behavior, stage at presentation and mortality 2,20. These warrants evaluating therapeutic options available to patients in real world settings 4,21,23. Thus, we sought to compare the efficacy of the two types of orchidectomy in terms of biochemical response in patients of African descent in our country.

## Methods

A prospective, single blind randomized and comparative hospital-based study among all consecutive advanced prostate cancer patients presenting to the Urology Division of a tertiary hospital, North Central, Nigeria over a period of 12months.

### Sample Size Estimation

Sample size was determined with the formula: n= [A+B] 2 ×2 × SD2 / DIFF2 where n= the sample size required in each group (double this for total sample); SD= standard deviation of primary outcome variable from previous study; DIFF= size of difference of clinical importance; A= significance level; B= documented mean power of 80% from previous study Values were deduced from a previous local study by Magoha (24), who assessed subcapsular orchidectomy in management of prostate cancer in Nigerians.

Hence, n= [1.96+0.84]2 ×2×212/14.62 = 32.4 approximately 32 patients and the total sample size was 2n=64 patients.

### Inclusion Criterion

Consenting patients with diagnosis of advanced prostate cancer that chose bilateral orchidectomy as mode of treatment.

### Exclusion Criteria

Patients excluded were those other histo-pathological types of prostate cancer, patients on neo-adjuvant hormonal therapy during the study period, patients with debilitating co-morbidities and patients with synchronous tumors.

### Ethical consideration

Ethical clearance was obtained from the hospital Ethical Review Committee (ERC) for a period of one-year. Informed consent was obtained from all patients enrolled in the study. Patient refusal to participate or desire to withdraw participation at any stage of the study was respected without attempt at coercion or inducement. Strict confidentiality was adhered to in the management of patient records, results and details.

### Patient selection and randomization

The patients recruited for the study were randomized to either of the two procedures designated as A and B in equal numbers using block randomization to limit bias and achieve even distribution between the treatment arms. Group A patients had bilateral sub-capsular orchidectomy (BSCO) while Group B patients had bilateral total orchidectomy (BTO).

A block size of four (4) was used because of the estimated sample size and the need to achieve an even balance between the groups. Each block contained an even number of four (4) procedures equally distributed but in different order; i.e., by random permutation.

The recruitment of patients for the study ended after attaining the estimated sample size.

### Peri-operative Assessment

All recruited patients had thorough clinical evaluation from the onset with detailed history and physical examination followed by appropriate pre-operative investigations such as complete blood count, fasting serum glucose and renal function test.

### Surgical Procedure

Procedures were carried out in supine position after administering pre-incision testicular nerve block (spermatic cord) with 1% lidocaine. This entails palpating and picking the spermatic cord between the thumb and index finger about 1cm infero-medial to the ipsilateral pubic tubercle. A fine needle (size 23G) was used to inject the 1% lidocaine to the overlying skin (wheal) and then further down into the cord. Care was taken to aspirate each time before injecting anaesthetic agent to avoid intravascular delivery. The proposed line of skin incision was also infiltrated with 1% lidocaine. These procedures were performed by the lead surgeon and trained senior registrars in Urology Division.

### Subcapsular Orchidectomy 25

A longitudinal median raphe scrotal incision was made after grasping the scrotum behind the testes to tense the overlying skin. The incision was deepened through the dartos muscle and cremasteric layers in each hemi-scrotum down to the tunica vaginalis. Each testis was delivered into the wound after opening the tunica vaginalis. The tunica albuginea was incised along the entire length of the anterior border and a piece of gauze used in sweeping out the testicular tissue from the edges to the center. A 3/0 vicryl suture was used to ligate the scooped tissue at the hilus before excision. The inner surface of the tunica albuginea as well as the base was fulgurated to destroy residual interstitial cells, and to control bleeding. Repair of the tunica albuginea was done with continuous 2/0 vicryl suture. Cauterization of bleeders in the dartos was done before closure with continuous 3/0 vicryl suture. Skin closure was achieved with 3/0 nylon vertical mattress sutures. Scrotal dressing was firmly applied and left in situ for 48hrs before release and wound inspection.

### Total Orchidectomy 25

Median raphe scrotal skin incision was made to deliver the testis into the wound from each hemi-scrotum and to expose the epididymis and the cord. The vas deferens was bluntly dissected from the spermatic vessels to ligate separately due to its blood supply. It was double clamped proximally and single clamped distally before dividing. It is doubly ligated with 3/0 vicryl suture. The rest of the cord was separated into two or three parts, doubly clamped, divided between clamps and ligated distally and proximally with 3/0vicryl. Cauterization of bleeders in the dartos was done before closure with continuous 3/0 vicryl suture. Skin closure was achieved with 3/0 nylon vertical mattress sutures and scrotal dressing firmly applied for 48hrs before release of pressure scrotal dressing.

### Data collection and analysis

A structured proforma was use to collect socio-demographic data, Gleason's score, testosterone and prostate specific antigen (PSA) for each patient. Blood samples for testosterone and PSA for biochemical assays were first drawn on the morning of surgery and ended at the third month of follow-up.

The data obtained were analysed using Statistical Package for Social Science “IBM SPSS version 20.0” Armonk, NY: IBM Corp. Mean and range were determined for variables with normal distribution while median and interquartile ranges were determined for non- parametric variables. The pre-operative and post -operative testosterone and PSA values were compared between and within the groups using Mann-Whitney-U test and paired sample t-test respectively based on the socio-demographics and Gleason's score using Chi square. The results were displayed using tables and graph. For all statistical tests, p < 0.05 was considered significant.

## Results

Sixty-four patients were recruited for the study (32 patients in each group). Mean age was 70.84±8.18years with a range of 49 to 90 years and the. majority of the subjects (93.8%) were married (Table 1). Comparison of the socio-demographic characteristics of the two study groups is as shown in Table 2.0. Mean age in BTO and BSCO groups were 71.63 ± 7.56 years and 70.06 ± 8.79 years respectively (p = 0.449). The mean Gleason's score of all subjects was 7.38 (±1.35) with a range of 4 to 9. The Gleason score of the tumor differentiation revealed that 50.0% were poorly differentiated (≥8), 29.7% were moderately differentiated (7) and 20.3% were well differentiated (≤6). The mean Gleason's score was 7.44±1.41 vs. 7.31±1.31 in the BTO and BSCO group respectively (p =0.714).

**Table 1:** Socio-demographic characteristic s of subjects

Variable	Frequency (n=64)	Percent
**Age**		
< 50	1	1.6
50 – 59	5	7.8
60 – 69	18	28.1
70 – 79	29	45.3
≥ 80	11	17.2
**Marital Status**		
Married	60	93.7
Divorced	1	1.6
Widowed	3	4.7

SD: Standard deviation

**Table 2:** Comparison of socio-demographic characteristics

Variable	Treatment			χ^2^	*p*- value
	BTO	BSCO	Total		
	n=32 (%)	n=32 (%)	N=64		
**Age**					
< 50	0 (0.0)	1 (3.1)	1		
50 – 59	3 (9.4)	2 (6.3)	5		
60 – 69	7 (21.9)	11 (34.4)	18		
70 – 79	15 (46.9)	14 (43.8)	29		
≥ 80	7 (21.9)	4 (12.5)	11	0.864^Y^	0.929
**Marital** **Status**					
Married	31 (96.9)	29 (90.6)	60		
Divorced	0 (0.0)	1 (3.1)	1		
Widowed	1 (3.1)	2 (6.3)	3	0.017^Y^	0.992

χ^2^: Chi square

Y: Yates correction (to reduce error in approximation

The median testosterone level of all subjects pre-operatively was 475ng/dl. The lowest testosterone value of 27ng/dl was recorded at 48hours post operatively, though castrate level (<50ng/dl) was already attained at 6hours post operatively. The mean pre-operative level of serum PSA among the 64 subjects was 59.82 ± 29.22ng/ml.

### Comparison of testosterone values between BTO and BSCO

**Figure 1:**
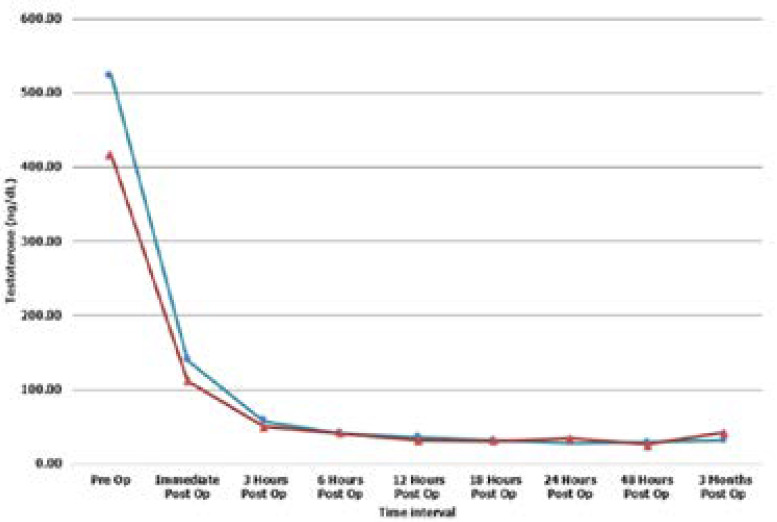
Relationship of Serum testosterone changes of bilateral total orchidectomy (BTO) and Bilateral Subcapsular orchidectomy (BSCO).

Using median values of testosterone assays due to skewed data, the pre-operative values of BTO and BSCO were favourably compared with no statistically significant difference (525.00ng/dl vs. 417.50ng/dl respectively, p = 0.515). The post-operative values showed no significant differences between the groups throughout the course of measurement as depicted in Table 3. Graphical illustration shows decline in serum testosterone from the onset of surgical castration till the 3rd post-operative month between the groups (Figure 1).

**Table 3:** Comparison of pre- and post-operative serum testosterone between the two treatment groups

Variable	Treatment		U	*p* value
	BTO	BSCO		
	n=32	n=32		
**Pre-Operative**				
Median (IQR)	525.00 (201.50–757.50)	417.50 (202.50–760.00)	463.50	0.515
**Immediate Post Op**				
Median (IQR)	139.50 (55.00–330.00)	111.50 (64.25–312.50)	506.50	0.941
**3 Hours Post Op**				
Median (IQR)	57.50 (30.50–97.50)	50.50 (28.50–112.00)	510.00	0.979
**6 Hours Post Op**				
Median (IQR)	41.00 (21.00–88.75)	41.50 (23.25–63.75)	509.50	0.973
**12 Hours Post Op**				
Median (IQR)	35.00 (21.25–58.75)	31.50 (17.90–53.75)	490.00	0.767
**18 Hours Post Op**				
Median (IQR)	31.00 (12.75–54.50)	30.00 (23.50–45.00)	485.50	0.722
**24 Hours Post Op**				
Median (IQR)	28.00 (10.50–48.75)	34.50 (23.25–48.75)	477.00	0.382
**48 Hours Post Op**				
Median (IQR)	29.00 (10.50–57.75)	26.00 (17.25–40.75)	511.00	0.989
**3 Months Post Op**				
Median (IQR)	31.50 (10.50–60.00)	42.00 (15.13–90.00)	433.50	0.291

†BTO: Bilateral total orchidectomy; BSCO: Bilateral subcapsular orchidectomy; Post Op: Post-operative; U: Mann- Whitney U; IQR: Interquartile Range; *p* value < 0.05.

### Comparison of PSA values between BTO and BSCO

The pre-operative values of serum PSA were compared between the BTO and BSCO group with mean values of 62.41ng/ml vs. 57.23ng/ml respectively. The difference was not significant at p value of 0.482 (Table 4). However, the post-operative values differ significantly at the 2nd month (27.45±21.72ng/ml vs. 13.73±12.72ng/ml respectively with p value = 0.003. There was a subsequent convergence at the 3rd month after surgery.

**Table 4:** Comparison of pre- and post-operative serum prostate specific antigen between the two treatment groups

Variable	Treatment		D	t	*p* value
	BTO	BSCO			
	n=32	n=32			
**Pre-Operative**					
Mean ± SD	62.41 ± 26.84	57.23 ± 31.63	5.18	0.707	0.482
**1 Month Post Op**					
Mean ± SD	30.03 ± 23.30	22.48 ± 18.63	7.55	1.432	0.157
**2 Months Post Op**					
Mean ± SD	27.45 ± 21.72	13.73 ± 12.72	13.72	3.083	0.003*
**3 Months Post Op**					
Mean ± SD	23.21 ± 22.91	22.91 ± 22.40	0.29	0.053	0.958

† BTO: Bilateral total orchidectomy; BSCO: Bilateral subcapsular orchidectomy; Post Op: Post-operative; t: Independent sample t-test; D: Mean difference; *p* value < 0.05.

## Discussion

The two group of treatment were comparable in terms of age (p = 0.449) and degree of differentiation of the adenocarcinoma (p =0.714). This represents a better match compared to a previous study by Zhang et al 13, in which the BSCO group had more poorly differentiated tumors. Prostate specific antigen (PSA) values which are useful in assessing prostate cancer were assessed pre- and post-orchidectomy serially. The mean pre-operative serum PSA value of 59.82±29.22 ng/ml using ELISA technique observed among the subjects. This value was beyond the expected value of <4ng/ml in healthy adult. However, this does not foreclose the fact that low serum PSA have been associated with non-palpable but clinically significant prostate cancer and high Gleason score 27,28.

Comparison of the pre-operative serum PSA values between the BTO and BSCO group yielded mean values of 62.41ng/ml and 57.23ng/ml respectively. The difference observed was not statistically significant and was in keeping with the extent of tumour differentiation as demonstrated by the mean Gleason's score of 7.44 and 7.31 for BTO and BSCO respectively. This high value of serum PSA in recruited patients was also noticed by Magoha in Nairobi 29 where 92% of patients with advanced prostate cancer had PSA >20ng/ml.

Following orchidectomy (all 64 subjects), a significant drop in serum PSA was observed till the 2nd month when mean value of 20.59±18.96ng/ml was recorded. This was followed by a slight rise in mean value to 23.06±22.48ng/ml in the third month. This sharp drop in serum PSA was more pronounced with the BSCO group in the 2nd month (p=0.003). However, the trend was not sustained into the 3rd month. On the contrary, a gradual but consistent decline of serum PSA was observed for BTO group throughout the course of measurement. It can be inferred that the slight rise in serum PSA (all 64 subjects) noticed in the 3rd month was contributed by those patients that had BSCO.

The role of serum PSA as a biomarker for prostate cancer diagnosis, prognosis, treatment monitoring as well as survival evaluation is well established 30–32; thus, the decline in PSA noted in this study was expected after orchidectomy due to loss of androgen trophism 33. However, the extent of decline and interval it takes to get to the nadir level was variable 32, depending on the death of differentiated neoplastic cells and/or decrease in expression of androgen receptor (AR) stimulated PSA in surviving tumour cells 30,34. It may be pre-mature to conclude that the PSA nadir in index study was attained at the 2nd month after surgery or that the slight rise noticed in the 3rd month was evidence of biochemical progression due to relatively short period of follow-up when compared to similar study with mean/median follow-up period of 28months and 35months 13,18. Albeit, it is common to find patients with advanced prostate cancer who undergo ADT having serum PSA nadir level of ≤ 4ng/ml at 6/7months post- surgery 30,32,33. This difference in pattern in relation to the BSCO group may be attributed to the heterogenous nature of prostate cancer clones of cells. These cells demonstrate variable degree of response to loss of androgen stimulus 20. However, this comparative difference was not reported in previous studies 13,18 and may require a larger randomized clinical trial to validate. The second biochemical parameter studied was serum testosterone whose median pre- operative value determined using ELISA was 475ng/dl (all 64 subjects). This pre-castrate value falls within the range expected for normal males 35. This was comparable to values obtained in previous studies 35–38 and did not differ between the two groups(p=0.515). The post-operative serum testosterone median values measured serially from 0 hour (immediately after removal of the testes) showed an initial steep decline from 475mg/dl to125mg/dl. This rapid drop excludes the possibility of surgical maneuver causing a temporary spike in the serum level testosterone. Similar precipitous decline was noted by Arcadi^39^ in fivof the six patients that had sub- capsular orchidectomy with serial monitoring of serum testosterone values. The post-operative changes based on surgical technique employed revealed that at 0 hour (i.e, immediately after orchidectomy), there was an approximately 74% decline in the pre-operative serum testosterone to produce median values of 189.5ng/dl and 11.5ng/dl for BTO and BSCO respectively. Similar trend was observed in successive measurements done at variable interval over the initial 48hours post-orchidectomy and at the 3rd month of follow-up. There was no significant difference but a near perfect alignment from the 3rd hour after surgery to the 3rd month of follow-up was observed.

Attaining castrate testosterone level is the aim of ADT and this level is defined as <50ng/dl or <5% of pre-castrate level though a stricter definition puts it at <20ng/ml 8,38. Analysis based on the technique of surgery showed that patients randomized into BTO and BSCO all attained the castrate serum testosterone level at 6hours post orchidectomy with median values of 41.0ngdl and 41.5ng/dl respectively(p=0.973). It can be inferred that both surgical procedures attained similar levels of hormonal ablation.

The lowest median testosterone value was at 48hours post-surgery(27.0ng/dl) but subsequently rose to 36.0ng/dl at the 3rd month of follow-up. This rise may be due to extra-testicular sources of androgens, especially, since the patients were not on maximal androgen blockade. The rise in median serum testosterone level may also be contributory to the increase in serum PSA value at the 3rd month after surgery. Specifically, at the 3rd month of follow-up, the median serum testosterone level was 31.5ng/dl and 42.0ng/dl for BTO and BSCO respectively. In absolute terms, these values represent a rise from the measurement at 48hours post-orchidectomy, with a higher percentage increase recorded in the BSCO group. Comparatively however, these changes were statistically not significant and as such cannot be conclusively implicated in the rise in mean serum PSA observed in the 3rd month of follow-up among subjects that had BSCO.

Furthermore, the linear graph comparison between the groups showed a near perfect alignment from the 3rd hour post-surgery till the 3rd month post orchidectomy and there was no significant difference in the recorded values throughout the course of measurement. This finding corroborates earlier studies 17,36,37 that disputed the possibility of persistence of ‘residual Leydig like cells’ in patients that had BSCO as claimed by O'Conor et al 16.

## Future research

The goal of treating patients with advanced prostate cancer is palliative and this is to maintain as best possible patients' quality of life and psychological well-being. That can include the cosmetic appeal of their scrotum post-surgery. Therefore, beyond the biochemical and clinical efficacy of two methods of surgical androgen deprivation, it is important to assess the psychological impact of each procedure on the patients within a randomized trial.

## Conclusion

This study observed that both procedures were effective in androgen deprivation with regards to attainment of castrate testosterone level and decline in the post-operative serum PSA. In comparing the two procedures by outcomes, the study showed that the biochemical responses to BTO and BSCO were similar and comparable.
